# Barriers to Antiretroviral Medication Adherence in People Living with HIV (PLHIV) at the Time of the COVID-19 Pandemic in the Philippines

**DOI:** 10.3390/tropicalmed8100461

**Published:** 2023-09-29

**Authors:** Philip John M. Joves, Melgar O. Matulac, Rodolfo S. Pagcatipunan

**Affiliations:** 1 Department of Internal Medicine, Adventist Medical Center Manila, Pasay City 1302, Philippines; doc_megoy@yahoo.com (M.O.M.);; 2 Department of Internal Medicine, Pasay City General Hospital, Pasay City 1302, Philippines; 3 St. Luke’s Medical Center Global City, Taguig 1634, Philippines

**Keywords:** HIV, Philippines, people living with HIV, AIDS, antiretroviral therapy, medication adherence, COVID-19

## Abstract

*Background:* During the COVID-19 pandemic, the HIV crisis in the Philippines worsened and triggered a chain reaction that disrupted the provision and utilization of HIV services. This study aims to fill in the gap in knowledge by exploring the possible association between sociodemographic characteristics and the barriers to ART adherence for PLHIV in the Philippines at the time of the COVID-19 pandemic. *Methods:* A cross-sectional study was performed by using a survey questionnaire, which was distributed via online social media (Twitter). Data were analyzed using the Stata software. *Results:* There is a significant association between the following treatment barriers and sociodemographic characteristics: the location of treatment hubs and respondents who finished college/graduate studies; checkpoints and crossing borders; and (1) respondents from Northern Luzon Region, (2) unemployed respondents and financial assistance—1. respondents 18 to 25 years old; 2. unemployed respondents—(3) respondents who finished elementary/high school and psychosocial support—(1) respondents from the NCR; (2) respondents 26 to 30 years old, stocks of ARVs and other medicines, and employed respondents. *Conclusions:* The results suggest a necessity for innovative approaches to make HIV care services, particularly ART, more accessible to PLHIV during the COVID-19 pandemic. Future large-scale studies exploring the association between sociodemographic characteristics and barriers to medication adherence of PLHIV during the COVID-19 pandemic are recommended.

## 1. Introduction

COVID-19 has caused over 760 million cases and recorded 6.9 million deaths since December 2019. Apart from the spread of the disease itself, the COVID-19 pandemic has had far-reaching effects. For example, several scheduled elections in 2020 were postponed, causing a negative impact on the democratic processes of numerous countries [1]. In addition, around 81 million jobs were lost because of COVID-19 in 2020 in the Asia Pacific region alone [2]. These negative consequences of the pandemic have affected the lives of those who are already vulnerable and marginalized pre-COVID-19. In turn, the pandemic has magnified existing gaps and inequalities in society, including access to basic social services such as healthcare [3].

In the Philippines, there have been 4,110,205 confirmed cases of COVID-19 with 66,661 deaths as of 6 September 2023 [4]. The COVID-19 pandemic compelled the Philippines to impose numerous community quarantine measures aimed at curbing the transmission of the novel coronavirus, consequently constraining mobility across the nation. This constraint in social mobility has in turn given rise to a diverse set of obstacles that hinder the community’s ability to access essential HIV prevention, testing, and treatment services [5].

HIV continues to be a major public health issue globally with an estimated 37.7 million people living with HIV (PLHIV) in 2020 [4]. Prior to the COVID-19 pandemic, the Philippines experienced an HIV crisis. It had a rapidly growing HIV epidemic in the Western Pacific region between 2010 and 2017, where a 174% increase in HIV incidence was noted. Numerous cultural and healthcare system factors have been identified as barriers to addressing the HIV crisis in the Philippines. To begin with, there is a prevailing HIV stigma deeply rooted in this society, associating HIV infection with sin and immorality. Second, there is a lack of access to medications for both HIV prevention and treatment. HIV pre-exposure prophylaxis (PrEP) is limited in Manila, and the only fixed-dose combination antiretroviral therapy accessible in the Philippines is lamivudine tenofovir-efavirenz (LTE). Lastly, the availability and delivery of services are unevenly distributed across the country, with HIV treatment centers predominantly concentrated in major urban areas like the National Capital Region [6].

During the COVID-19 Pandemic, the HIV crisis in the Philippines worsened. The national prevention package received by males who have sex with males (MSM) and transgender women (TGW), defined as having received HIV information, with access to condoms whether free or bought, and having been tested for HIV in the past 12 months, was reduced from 26% to 17%. Numerous testing and outreach modalities such as community-based screening, outreach testing, and facility testing were disrupted because of community restrictions, which led to a 61% decrease in HIV testing in 2020 compared with 2019, as reported by the Philippines’ Department of Health (DOH). According to the HIV, AIDS, and ART Registry of the Philippines (HARP), following the decline in testing, there has been a 37% decrease in the number of newly diagnosed cases in 2020 compared with 2019. Additionally, there was an observed 28% decrease in treatment initiation and a 19% increase in the treatment gap amid the pandemic [5].

The COVID-19 pandemic triggered a chain reaction that disrupted the provision and utilization of HIV services. The decrease in prevention coverage consequently affected the use of condoms among MSMs (males who have sex with males) and TGW (transgender women). This, in combination with continued high-risk behavior and a larger treatment gap for 2020, the rate of new HIV infections doubled from 10% between 2019 and 2020 to 21% between 2020 and 2021 [5].

Previous studies worldwide have reported barriers that can impact or hinder access to HIV treatment and care services among PLHIV. These encompass the limited availability of HIV care services, an insufficient number of qualified healthcare professionals to deliver these services to people, long-distance travel to healthcare facilities or HIV clinics, the absence of convenient public transportation options, poor health literacy, shortages in the quantity of ARV medicines, and the unaffordability of HIV care-service-related costs and the inability of PLHIV to afford them [7,8,9,10,11,12,13,14,15,16,17]. Many of these barriers, such as the unavailability of public transportation and the cost of courier services for ART delivery, have also been encountered by PLHIV in various regions of the Philippines [3].

This study aims to fill in the gap in knowledge by exploring the possible association between sociodemographic characteristics and barriers to ART adherence for PLHIV at the time of the COVID-19 pandemic in the Philippines in order to develop new strategies and modify existing services to address the needs of PLHIV.

## 2. Materials and Methods

### 2.1. Study Design

A cross-sectional study was conducted by using a survey questionnaire (Appendix A), which was distributed via online social media (Twitter). Twitter was chosen as the source of participants since it serves as a safe avenue for participants to disclose their sexual identities. Participants are able to discuss aspects of their personal lives, such as romances and lifestyles, more openly and freely on Twitter, and this is the reason the questionnaire was posted on it [18].

The survey questionnaire used in this study was the same questionnaire used by Engr. Xavier Javines Bilon in his published report entitled “Leaving No One Behind: Treatment and Care Concerns of People Living with HIV in the Time of COVID-19—A Philippine Situationer”, which was validated by UNDP and UNAIDS. It was divided into five parts. The first part consists of an introduction regarding the purpose of the research. The second part includes the respondents’ consent to voluntarily participate in the research. The third part of the questionnaire includes the unique identification code of each respondent, which was patterned in RITM when enrolling HIV patients for ARV treatment.

The fourth part of the questionnaire is the sociodemographic questions, which include the age, region of residence, gender identity, sexual orientation, educational background, and employment status, self-reported by all included participants. The fifth part of the questionnaire consists of one close-ended question on issues encountered by the respondent in accessing antiretroviral therapy in the time of COVID-19 and two open-ended questions where the respondent can provide more details regarding the issues they identified in the previous question and other HIV-related concerns that they have. Specifically, these are the questions asked: (1) “What issues did/do you encounter in accessing HIV treatment and care services, including antiretroviral therapy, in the time of COVID-19? Check all that apply.”; (2) “Please provide more details regarding the issues you identified in the previous question.”; and (3) “You may also share other HIV-related concerns that you currently have” [3].

### 2.2. Sample Size Computation

The survey questionnaire accepted responses from 26 November 2021 to 10 January 2022. We received 118 responses with 2 duplicates. After processing, we had a non-probability sample of 116 valid responses (*n* = 116). The computed sample size was 82 using the Open Epi software version 3.01. The following assumptions were used:

- 95% confidence level;
- Expected % of barriers (UNDP and UNAIDS, 2021):
  ○ 59%—location of treatment hubs;
  ○ 57%—checkpoints and crossing borders;
  ○ 54%—stock of ARVS;
- 20% of estimate relative precision.

### 2.3. Data Analysis

Data were analyzed using the Stata software version 15. Categorical variables were summarized using frequencies and percentages; quantitative data were summarized using mean and standard deviation. The chi-square test or Fisher’s exact test, whichever was more appropriate, was used to determine the association between sociodemographic characteristics and HIV treatment barriers (Table 1).

## 3. Results

### 3.1. Sociodemographic Characteristics

**Figure 1:**
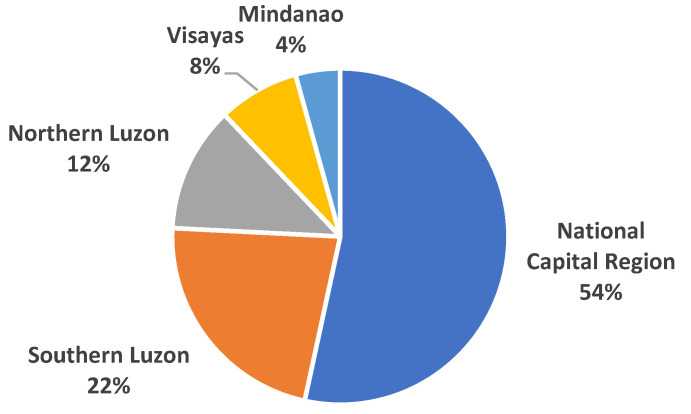
Distribution of PLHIV by region.

**Figure 2:**
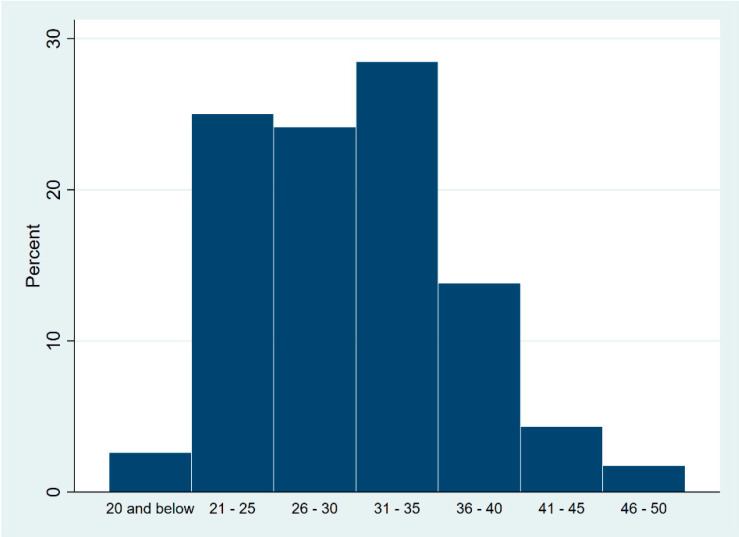
Distribution of PLHIV by age group.

A total of 116 respondents answered the online survey. The majority (53.4%) were from the NCR, 34.5% were from northern and southern Luzon, and 12.1% were from Visayas and Mindanao (Table 2 and Figure 1). The Study participants were 18 to 50 years old (Figure 2) with a mean age of 30.25 years old (SD = 6.22), and 91.3% were 21 to 40 years old. Respondents were mostly males (95.7%), were either homosexual (59.5%) or bisexual (37.9%), had college or graduate degrees (87.1%), and were employed (70.7%).

### 3.2. Identified HIV Treatment Barriers

The most common HIV treatment barriers reported by the respondents in accessing treatment and care were the unavailability of transportation and the cost of courier services for ARV delivery (62.1%), the location of treatment hubs (52.6%), and financial assistance (37.9%). The least frequent barriers to HIV treatment were temporary shelter and housing (6%) and verification, including ARV booklets (9.5%). Around 40% of the respondents reported having three or more issues in HIV treatment, and two respondents, both from the NCR, were experiencing all eight issues (Table 3).

### 3.3. HIV Treatment Barriers and Respondents by Region

**Figure A1:**
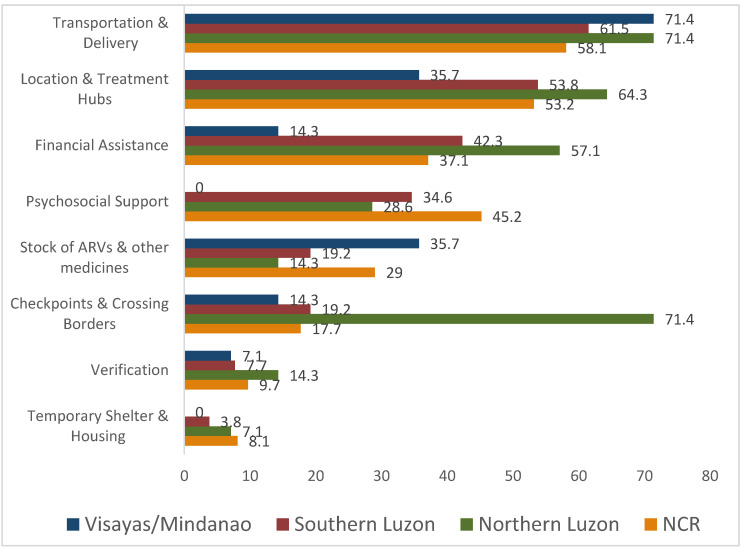
Percentage of PLHIV by type of issue and region.

Tests for association showed that that there were significant associations between regions, checkpoints and crossing borders, and psychosocial support (Table 4 and Figure A1). Issues with checkpoints and crossing borders were significantly higher in Northern Luzon (71.4%) than in other (17.7%) regions (*p* < 0.001).

On the other hand, issues with psychosocial support were significantly higher (*p* = 0.018) in the NCR (45.2%) compared with other regions (24.1%). Two of the respondents who left their families in the province to work in the city said that life was hard because they had no support group and were far away from home:

“*I guess lack of people or group of people that would check on you. to be honest, it’s really hard to find a support or friend that share the same situation just like mine. I know I could find them from maybe institution from a hub or even here on twitter, but, it’s really hard. I’ve been alone since I was diagnosed*”

(Participant 1)

“*It’s kind of hard specially since I am living alone here in the metro and I am having trust issues with some of my friends about my status*”

(Participant 2)

### 3.4. HIV Treatment Barriers and Respondents by Age Group

**Figure A2:**
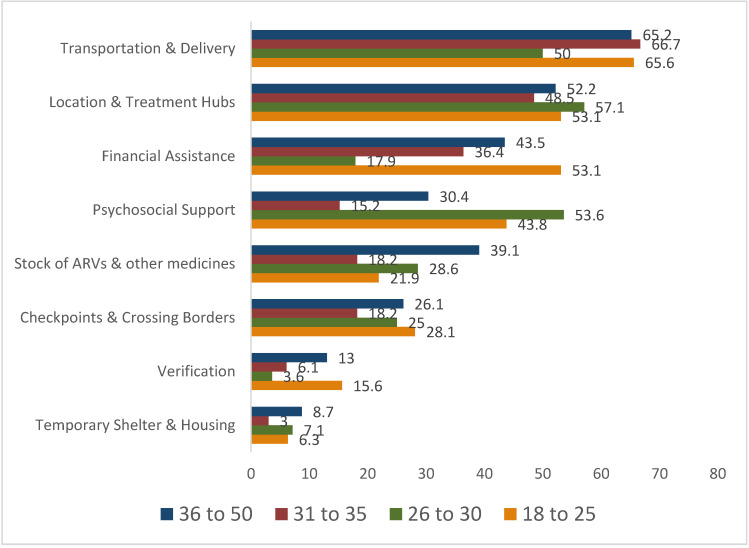
Percentage of PLHIV by type of issue and age group.

There was a significant association between issues with financial assistance and respondents aged 18 to 25 years old (53.1%, *p* = 0.041)) (Table 5 and Figure A2). Two of the respondents expressed their dilemmas as students regarding how they were going to support themselves financially to afford transportation to their treatment hubs.

“*During the Enhanced Community Quarantine, public transportations were banned so the only way to get ARVs is through Grab Express, or Lalamove. I’m only a student without savings that time so payment for those services were kind of expensive for me*”

(Participant 3)

“*The treatment hub is far from the place where I am staying. I’m only a student, that is why commuting is one of the hindrances why I cannot go regularly at the treatment hub to have for my check-up every 6 months*”

(Participant 4)

### 3.5. HIV Treatment Barriers and Respondents by Educational Status

**Figure A3:**
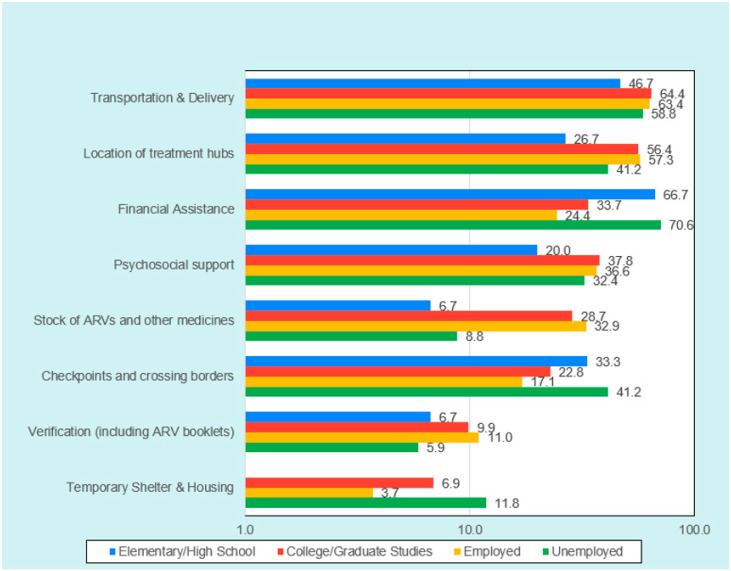
Percentage of PLHIV by type of issue, educational status, and employment status.

There was a significant association between respondents who finished elementary or high school (66.7%, *p* = 0.014) and issues with financial assistance (Table 6 and Figure A3). Conversely, issues with the location of treatment hubs were significantly higher (*p* = 0.031) among those who finished college or graduate studies (56.4%). A respondent who was initially enrolled at Pasay City had to transfer to another treatment hub nearer to his home address, while another respondent reported that he had difficulty obtaining a refill because his workplace has a “No work, no pay” policy:

“*During Enhanced Community Quarantine, my hub is located in Pasay. It was hard to coordinate to the hub located in Cavite because it is not my primary hub. Also, my family doesn’t know that I have it thus it was hard to do the paper works and they ask what is the delivery is for. And since the Cavite Hub is not my primary hub, I sometimes get 2 bottles only*”

(Participant 5)

“*All kinds of transportation have been shut off during the lockdown. The location of the treatment hub is quite far. During lockdown, I don’t get paid if I don’t go to work*.”

(Participant 6)

### 3.6. HIV Treatment Barriers and Respondents by Employment Status

Those who were unemployed had significant association issues with checkpoints and crossing borders (41.2%) and financial assistance (70.6%) compared with those who were employed (17.1% and 24.4%, respectively) (Table 7 and Figure A3). One of the respondents reported that his treatment hub had no advice as to how he should obtain his ARV refill. He had to research on his own on the internet for possible options since calling the contact number of his treatment hub was impossible. On top of that, because of financial constraints and the COVID-19 lockdown, he had to travel by foot to visit his treatment hub.

“*During lockdowns, you have to walk to the nearest hospital. You’ll encounter checkpoints that asked you questions if you’re the one allowed to travel to get certain items including medications. Since you’re too far from the hub, you have to get your medication to the nearest hospital hoping you’ll get one. The delivery is much trickier because you don’t have any idea what to do that’s why you have to search the net for information if it’s possible. Calling the hub was impossible back then I guess the phone to the department was busy too. Money was also a problem since my siblings stop working and its troubling time*.”

(Participant 7)

However, stocks of ARVs and other medicines were more problematic (*p*-0.007) among those who were employed (32.9%) than those who were unemployed (8.8%). One of the respondents said that he needed to make an excuse to travel and receive his ARV refill, while another respondent had difficulty finding time to get tested for CD4 count and viral load given his work schedule:

“*I do live miles away from my treatment hub. I am employed and need to get excused to travel every refill time*”

(Participant 8)

“*My work schedule is somehow a hindrance to go. This makes it hard for me to do testing for Viral Load and CD4 count*”

(Participant 9)

“*All kinds of transportation have been shut off during the lockdown. The location of the treatment hub is quite far. During lockdown, I don’t get paid if I don’t go to work*.”

(Participant 10)

## 4. Discussion

PLHIV have a high probability of experiencing treatment interruptions because of restrictions on non-emergency medical appointments related to social distancing COVID-19 protocols. In turn, those who are not taking ART or whose disease is not well managed may be at increased risk of acquiring COVID-19 because of compromised immune systems, leading to serious symptoms and death [19,20,21,22,23,24,25,26,27,28,29].

A number of barriers in previous studies pre-pandemic were identified that are consistent with our findings. These included the location of treatment hubs, financial barriers, stock of ARVs, checkpoints and crossing borders, and psychosocial support [7,8,9,10,11,12,13,14,15,16,17].

In this study, the respondents reported that the top three issues encountered in the Philippines during the time of the COVID-19 pandemic were transportation and delivery, the location of treatment hubs, and financial assistance. The first and second issues were also cited as common barriers encountered by PLHIV in accessing HIV treatment and care in an existing study in the context of the Philippines [3]. Similarly, these barriers were also reported to hinder access to HIV treatment and care in other places (e.g., Indonesia and Africa) [30,31,32,33,34]. However, to the best of our knowledge, this is the first study to specifically explore the significant associations between sociodemographic characteristics and the identified barriers to the medication adherence of PLHIV in the Philippines during the COVID-19 pandemic.

### 4.1. Location of Treatment Hubs

There is a significant association between the location of treatment hubs and respondents who finished college/graduate studies. Respondents reported that they had difficulty visiting the treatment hub because of their work schedules and “no work, no pay” policies in the workplace. This was worsened by the location of their treatment hubs.

Some PLHIV in Metro Manila opted to use courier services such as Grab or Lala Move. The respondents experienced missed doses of daily ARV because of delays in delivery, ARV refills were delivered to the wrong address, addresses were out of coverage for the courier, or they had difficulty contacting their treatment hubs to inquire if there was an option for ARV delivery. This was worsened for those who also experienced financial constraints because of the delivery fee. In addition, some treatment hubs do not allow the delivery of ARV and require patients to personally claim it at the clinic.

In Mindanao, the situation is worse. One of the respondents received his ARV refill by having it delivered by a provincial bus en route to his location. Some respondents had to transfer to other places of residence nearer to their treatment facilities. Table 8 shows the top five farthest respondents living with HIV from an HIV facility.

This trend in difficulty reaching the location of treatment hubs for respondents due to the COVID-19 pandemic was observed to be happening similarly in Chinese and Ugandan studies [35,36].

### 4.2. Checkpoints and Crossing Borders

There is a significant association between checkpoints and crossing borders and respondents from Northern Luzon and unemployed respondents. Approximately 24% of respondents had to go to a neighboring city/municipality to get to the nearest HIV facility, which makes crossing borders and passing through checkpoints inevitable. PLHIV experience discomfort or anxiety in disclosing their HIV status at checkpoints to be allowed to enter the area where the nearest treatment hub is. Some respondents were worried that they might be misunderstood because of their health condition or, moreover, discriminated against when they disclosed their HIV status. These findings were also reported as a hindrance to medication adherence in some other countries [37,38].

### 4.3. Financial Assistance

There is a significant association between financial assistance and respondents aged 18–25 years old, unemployed respondents, and respondents who finished elementary/high school. The demand for blue-collar jobs decreased during the COVID-19 pandemic, mainly because of COVID-19 measures implementing social distance protocols. This, in turn, led to difficulty for respondents in landing white-collar jobs since the usual requirement is a college graduate. 

These negative economic impacts produced by the COVID-19 pandemic were reflected in the disruption of income-generating activities and reduced or lost incomes, which led to an inability to afford healthcare-related expenses for the respondents. This, in turn, may have long-term financial implications for already poor participants and their families, who experienced losses of income during the pandemic. These findings are in line with previous studies, which have reported financial hardship as one of the barriers to accessing ART and other treatment services for PLHIV [12,15,39].

### 4.4. Psychosocial Support

During the time of community quarantine, around 35% of respondents reported that they experienced anxiety and depression with no psychosocial support available since social gatherings or meetings were not allowed. Some PLHIV noted that they felt something was still lacking despite the support they received from their families. Meanwhile, some respondents looked for empathy from other PLHIV who faced the same battle. Fear and anxiety related to transmission are the common denominators of HIV and COVID-19. This pandemic caused PLHIV to have a high stress level because of the possibility of acquiring another virus that could kill them, in addition to the fact that there was physical distancing and social isolation recommended by the CDC to reduce the spread of COVID-19, the lack of knowledge on how SARS-CoV-2 may synergize with HIV and the absence of scientifically proven treatments to address symptoms and prevent death [40,41,42,43]. Pandemic fear in general can cause or worsen existing mental health issues such as anxiety, depression, and substance abuse, especially among elderly people with HIV. Older PLHIV confront a myriad of mental health issues such as depression, stigma, and ageism [44,45,46,47,48]. Social distancing and lockdowns in many countries caused a huge psychological impact, undermining the overall well-being of these individuals, who lost hope of improving their health outcomes, consequently leading to a decrease in adherence to ART [49,50,51,52,53,54,55].

### 4.5. Stock of ARV and Other Medicines

An estimated 26% of respondents reported the unavailability or limited availability of certain ARV drugs and other medicines in their respective treatment hubs within and outside Metro Manila. These drugs include cotrimoxazole, azithromycin, isoniazid, and nevirapine. Several respondents experienced partial refills wherein they were provided as few as 15 tablets or one or two bottles instead of the usual three bottles. One respondent had to shift to another combination of ARV drugs because of unavailability, shifting from lamivudine/nevirapine to tenofovir/lamivudine/dolutegravir. One respondent had difficulty with ARV refills since his treatment hub did not operate during weekends because of the pandemic, while one respondent reported that the treatment hub provided expired ARV drugs. Meanwhile, one respondent experienced being charged for prophylaxis medications that were previously free and came in packages with his ARV. The treatment hub informed him that this was due to a decrease in budget at the treatment facility. These issues are compounded by other issues, such as transportation, the location of HIV facilities, checkpoints and crossing borders, and financial assistance, which made HIV treatment and care services more inaccessible to PLHIV at the time of the COVID-19 pandemic.

Some respondents also had difficulty accessing ARV refills because they did not have their ARV booklets, which they were asked to provide by their treatment hubs. One respondent reported that his booklet was empty because there were no logs from the hub to keep track of his quarterly issued medications because of an inability to visit the clinic.

Many of the respondents suggested that viral load and CD4 count testing should come in packages together with their ARV treatments. In addition, some Filipinos living with HIV are still not fully educated about HIV and COVID-19. Several respondents were concerned about whether there are possible adverse reactions between COVID-19 vaccines and the ARV medications that they are taking.

### 4.6. Study Limitations and Strengths

All participants in the inclusion criteria were Filipino, 18 years old and above, and a confirmed case of HIV on maintenance of ARV. The limitation of this study in the aspect of data gathering is mainly due to the COVID-19 pandemic, in which close physical contact was prohibited and needed in order to maintain social distancing policies to avoid a rapid increase in cases, especially in immunocompromised respondents. As such, data collection was only conducted through the use of an online questionnaire distributed via Twitter. However, to the best of our knowledge, there has not been much evidence in the context of the Philippines about the association between sociodemographic characteristics and the identified barriers to adherence to ART. Thus, the current findings provide useful information for the government and healthcare systems and facilities in various regions of the Philippines, as well as other similar settings globally in order to develop strategies to support the accessibility of ART among PLHIV during the COVID-19 pandemic.

## 5. Conclusions

This study revealed that there is a significant association between the following treatment barriers and sociodemographic characteristics: the location of treatment hubs and respondents who finished college/graduate studies; checkpoints and crossing borders; and (1) respondents from Northern Luzon Region, (2) unemployed respondents and financial assistance—1. respondents 18 to 25 years old; 2. unemployed respondents—(3) respondents who finished elementary/high school and psychosocial support—(1) respondents from the NCR; (2) respondents 26 to 30 years old, stocks of ARVs and other medicines, and employed respondents.

The results suggest the necessity of innovative approaches in order to make HIV care services, particularly regarding ART, more accessible to PLHIV during the COVID-19 pandemic. This could involve implementing a community-based strategy by leveraging established networks of community health workers, a method that has demonstrated success in various countries [55]. Additionally, there should be coordinated efforts to offer ART at public health centers, which are conveniently located in every subdistrict. Future large-scale studies exploring the association between sociodemographic characteristics and barriers to medication adherence for PLHIV during the COVID-19 pandemic are recommended.

## Figures and Tables

**Table 1:** Sample size distribution by HIV treatment barriers.

Barriers	%	+/− Absolute Precision (20% of Estimate)	*n*
Transportation and delivery	67%	1.3	1419
Location of treatment hubs	59%	11.8	67
Checkpoints and crossing borders	57%	11.4	73
Stock of ARVs	54%	10.8	82
Financial assistance	41%	8.2	139
Psychosocial support	20%	4.0	384
Temporary shelter and housing	4%	0.8	2300
Overseas Filipino Workers’ (OFWs’) concerns	3%	0.6	3096

**Table 2:** Distribution of respondents by sociodemographic characteristics.

Characteristic	No.	%
*n*	116	100
**Region**		
National Capital Region (NCR)	62	53.4
Southern Luzon	26	22.4
Northern Luzon	14	12.1
Visayas	9	7.8
Mindanao	5	4.3
**Age Group**		
20 and below	3	2.6
21–25	29	25.0
26–30	18	24.1
31–35	33	28.4
36–40	16	13.8
41–45	5	4.3
46–50	2	1.7
**Gender Identity**		
Male	115	99.1
Female	1	0.9
**Sexual Orientation**		
Homosexual	69	59.5
Bisexual	44	37.9
Heterosexual	3	2.6
**Educational Status**		
Elementary/High School	15	12.9
College/Graduate Studies	101	87.1
**Employment Status**		
Employed	82	70.7
Unemployed	34	29.3

**Table 3:** Type and number of HIV treatment barriers.

Issues	No.	%
*n*	116	100
Transportation and delivery	72	62.1
Location of treatment hubs	61	52.6
Financial assistance	44	37.9
Psychosocial support	41	35.3
Stock of ARVs and other medicines	30	25.9
Checkpoints and crossing borders	28	24.1
Verification (including ARV booklets)	11	9.5
Temporary shelter and housing	7	6.0
**Number of Issues**		
1	34	29.3
2	34	29.3
3	24	20.7
4	11	9.5
5	7	6.0
6	3	2.6
7	1	0.9
8	2	1.7

**Table 4:** Association between HIV treatment barriers and respondents by region.

Barrier	NCR (*n* = 62)		Northern Luzon		Southern Luzon		Visayas/Mindanao		*p*-Value
	No.	%	No.	%	No.	%	No.	%	
*n*	62		14		26		14		
Checkpoints and crossing borders	11	17.7	10	71.4	5	19.2	2	14.3	<0.001 ^a^
Financial assistance	23	37.1	8	57.1	11	42.3	2	14.3	0.124
Location of treatment hubs	33	53.2	9	64.3	14	53.8	5	35.7	0.495
Psychosocial support	28	45.2	4	28.6	9	34.6	0	0	0.018 ^b^
Stock of ARVs and other medicines	18	29.0	2	14.3	5	19.2	5	35.7	0.456
Temporary shelter and housing	5	8.1	1	7.1	1	3.8	0	0	0.447 ^c^
Transportation and delivery	36	58.1	10	71.4	16	61.5	10	71.4	0.690
Verification (including ARV booklets)	6	9.7	2	14.3	2	7.7	1	7.1	0.939 ^d^

^a^ Fisher’s exact test between Northern Luzon (71.4%) and others (17.7%). ^b^ Chi-square test between NCR (45.2%) and others (24.1%). ^c^ Fisher’s exact test between NCR (8.1%) and others (3.7%). ^d^ Chi-square test between NCR (9.7%) and others (9.3%).

**Table 5:** Association between HIV treatment barriers and respondents by age group.

Barrier	18 to 25		26 to 30		31 to 35		36 to 50		*p*-Value
	No.	%	No.	%	No.	%	No.	%	
*n*	32		28		33		23		
Checkpoints and crossing borders	9	28.1	7	25.0	6	18.2	6	26.1	0.807
Financial assistance	17	53.1	5	17.9	12	36.4	10	43.5	**0.041**
Location of treatment hubs	17	53.1	16	57.1	16	48.5	12	52.2	0.927
Psychosocial support	14	43.8	15	53.6	5	15.2	7	30.4	**0.011**
Stock of ARVs and other medicines	7	21.9	8	28.6	6	18.2	9	39.1	0.321
Transportation and delivery	21	65.6	14	50.0	22	66.7	15	65.2	0.513
	18 to 30				31 to 50				
Temporary shelter and housing	4		6.67		3		5.36		1.000
Verification (including ARV booklets)	6		10.0		5		8.93		0.844

Note: All tests were performed using chi-square except for temporary shelter and housing, where Fisher’s exact test was used.

**Table 6:** Association between HIV treatment barriers and respondents by educational status.

Barrier	Elementary/High School		College/Graduate Studies		*p*-Value	Test
	No.	%	No.	%		
*n*	15		101			
Checkpoints and crossing borders	5	33.3	23	22.8	0.353	Fisher’s
Financial assistance	10	66.7	34	33.7	**0.014**	Chi-square
Location of treatment hubs	4	26.7	57	56.4	**0.031**	Chi-square
Psychosocial support	3	20.0	38	37.6	0.183	Chi-square
Stock of ARVs and other medicines	1	6.7	29	28.7	0.111	Fisher’s
Temporary shelter and housing	0	0	7	6.9	0.592	Fisher’s
Transportation and delivery	7	46.7	65	64.4	0.188	Chi-square
Verification (including ARV booklets)	1	6.7	10	9.9	1.000	Fisher’s

**Table 7:** Distribution of respondents by employment status per HIV treatment barrier.

Barrier	Employed		Unemployed		*p*-Value	Test
	No.	%	No.	%		
*n*	82		34			
Checkpoints and crossing borders	14	17.1	14	41.2	**0.006**	Chi-square
Financial assistance	20	24.4	24	70.6	**<0.001**	Chi-square
Location of treatment hubs	47	57.3	14	41.2	0.113	Chi-square
Psychosocial support	30	36.6	11	32.4	0.664	Chi-square
Stock of ARVs and other medicines	27	32.9	3	8.8	**0.007**	Chi-square
Temporary shelter and housing	3	3.7	4	11.8	0.192	Fisher’s
Transportation and delivery	52	63.4	20	58.8	0.643	Chi-square
Verification (including ARV booklets)	9	11	2	5.9	0.504	Fisher’s

**Table 8:** Top Five Farthest Respondents from an HIV Facility.

Residence of Client	Nearest HIV Facility	Distance	Travel Time by Walking
Zamboanga del Sur	Age—Healthily Wellness Hub	405 km	72 h
El Nido, Palawan	Ospital ng Palawan	262 km	48 h
Katico, Sultan Kudarat	Cotabato Regional and Medical Center	111 km	19 h
Moalboal, Cebu	Cebu City Health Center	88 km	17 h
San Joaquin, Iloilo	The Medical City Visayas	53 km	11 h

## Data Availability

The data cannot be shared publicly because they are confidential. The data are available from the Department of Internal Medicine of Adventist Medical Center Manila for researchers who meet the criteria for access to confidential data.

## Appendix A

SURVEY QUESTIONNAIRE

**I. Introduction**

Good day!

Thank you in advance for your interest in this research survey. This research aims to determine the barriers of adherence to antiretroviral therapy among people living with HIV (PLHIV) in the Philippines at the time of COVID-19 Pandemic. The participant is expected to answer all items completely and as honestly as possible.

This study will contribute to the development of programs by healthcare institutions and LGUs that will give added support to PLHIV.

All information obtained from each participant will be treated with utmost confidentiality. The data will be stored securely and only the researcher will have access to the information. If the study is eventually published or reported, no identifiable personal information of any participant will be included.

**II. Participant’s Agreement**

I am a Filipino, of legal age (18 to 60 years old), agree to participate in the research study conducted by the researcher of the Department of Internal Medicine, Adventist Medical Center Manila.

I am aware that my participation in this study is completely voluntary. If for any reason and at any time, I wish to cease my participation, I may do so without having to give any explanation. The researcher has reviewed the individual and social benefits of this project with me, and I understand the purpose of this research.

I am aware that the data gathered in this study will remain confidential and anonymous throughout the duration of the project.

I am hereby giving my consent and voluntarily participate in the data gathering of this research study:

○ Yes
○ No

**III. Identification Code**

Unique identifiers are needed to avoid duplicative counts for this research survey. Please type the first 2 letters of your father’s first name (e.g., Juan = JU), first 2 letters of your mother’s first name (e.g., Maria = MA), your sibling order (e.g., Eldest = 01, Second = 02, etc.) and your birthday (e.g., 13 June 2021 = 06132021).

Example Complete Unique Identifier Code: JU-MA-01-06132021

**IV. Socio-demographic Characteristics**

*Age (years)*

<20 years old
21–25 years old
26–30 years old
31–35 years old
36–40 years old
41–45 years old
46–50 years old
>51 years old

*Region of Residence*

National Capital Region
Northern Luzon
Southern Luzon
Visayas
Mindanao

*Gender Identity*

Male
Female

*Sexual Orientation*

Homosexual
Bisexual
Heterosexual

*Educational Background*

Post Graduate Studies
College
High school
Elementary

*Employment Status*

Employed
Unemployed

**V. Close and Open-ended Questions**

1. What issues did/do you encounter in accessing HIV treatment and care services, including antiretroviral therapy, in the time of COVID-19? Check all that apply.
  - Transportation and delivery
  - Location of treatment hubs
  - Checkpoints and crossing borders
  - Stock of ARVs and other medicines
  - Financial assistance
  - Employment
  - Psychosocial support
  - Verification (including ARV booklets)
  - Temporary shelter and housing
2. Please provide more details regarding the issues you identified in the previous question.
3. You may also share other HIV-related concerns that you currently have.
